# Intestinal Flora and Disease Mutually Shape the Regional Immune System in the Intestinal Tract

**DOI:** 10.3389/fimmu.2020.00575

**Published:** 2020-04-03

**Authors:** Bolun Zhou, Yutong Yuan, Shanshan Zhang, Can Guo, Xiaoling Li, Guiyuan Li, Wei Xiong, Zhaoyang Zeng

**Affiliations:** ^1^NHC Key Laboratory of Carcinogenesis, Hunan Cancer Hospital and the Affiliated Cancer Hospital of Xiangya School of Medicine, Central South University, Changsha, China; ^2^Department of Stomatology, Xiangya Hospital, Central South University, Changsha, China; ^3^Key Laboratory of Carcinogenesis and Cancer Invasion of the Chinese Ministry of Education, Cancer Research Institute, Central South University, Changsha, China; ^4^Hunan Key Laboratory of Nonresolving Inflammation and Cancer, Disease Genome Research Center, the Third Xiangya Hospital, Central South University, Changsha, China

**Keywords:** intestinal flora, intestinal tract, regional immune system, probiotic, fecal microbiota transplantation

## Abstract

The intestinal tract is the largest digestive organ in the human body. It is colonized by, and consistently exposed to, a myriad of microorganisms, including *bifidobacteria, lactobacillus, Escherichia coli, enterococcus, clostridium perfringens*, and *pseudomonas*. To protect the body from potential pathogens, the intestinal tract has evolved regional immune characteristics. These characteristics are defined by its unique structure, function, and microenvironment, which differ drastically from those of the common central and peripheral immune organs. The intestinal microenvironment created by the intestinal flora and its products significantly affects the immune function of the region. In turn, specific diseases regulate and influence the composition of the intestinal flora. A constant interplay occurs between the intestinal flora and immune system. Further, the intestinal microenvironment can be reconstructed by probiotic use or microbiota transplantation, functioning to recalibrate the immune homeostasis, while also contributing to the treatment or amelioration of diseases. In this review, we summarize the relationship between the intestinal flora and the occurrence and development of diseases as an in-turn effect on intestinal immunity. We also discuss improved immune function as it relates to non-specific and specific immunity. Further, we discuss the proliferation, differentiation and secretion of immune cells, within the intestinal region following remodeling of the microenvironment as a means to ameliorate and treat diseases. Finally, we suggest strategies for improved utilization of intestinal flora.

## Introduction

The intestine is the largest digestive organ in the human body and one of the largest organs in contact with the outside world. In addition to digesting food to facilitate the absorption of nutrients, it has a variety of other functions, including transmission of information and regulation of metabolism [1]. Due to its unique structure, the intestine is constantly exposed to various antigens and microbes. To protect the body from pathogens, while also maintaining a stable environment, the human intestinal tract has evolved unique regional immune characteristics maintained by the mature intestinal mucosal immune system [2]. This intricate system involves intestinal epithelial cells, and intestinal lymphoid tissue composed of Peyer's patches, isolated lymphoid follicles, mesenteric lymph nodes, etc. [3]. The congenital and adaptive immune mechanisms created by the unique structure, function, and microenvironment of the intestine differ from those of the central and peripheral immune organs, forming the regional immunity of the intestine. The intestinal flora also plays an important role in maintaining intestinal homeostasis. Further, the flora alters the structure and function of the immune system, reshaping the immune microenvironment, and promoting or interfering with development of specific diseases.

In fact, the immune function of the intestinal region directly affects the development of many intestine-specific diseases. However, the integrity of intestinal immune function depends on the expression of congenital genes and regulation of the neuroendocrine system [4, 5]. Additionally, the microenvironment created by the intestinal flora and its products are important factors affecting the immunity of the intestinal region. In early life, appropriate intestinal colonization by specific microflora stimulates maturation of the intestinal mucosa-associated lymphoid tissue [6]. Should the appropriate intestinal flora fail to form during this life stage, the function of the intestinal immune system becomes impaired, leading to increased incidence and/or morbidity of certain intestinal diseases, including ulcerative colitis, Crohn's disease, and colorectal cancer [7, 8]. In addition, activation of the immune system in the intestinal region depends on expression of pathogen-associated molecular patterns (PAMPs), e.g., intestinal microbial products. The intestinal flora secretes a variety of metabolites and bacteriocins as well as other PAMPs that activate the intestinal immune system via pattern recognition receptors (PRRs), and through adhesion to the epithelium, thus impacting the occurrence and development of disease [9]. Aside from the bacteria that colonize the intestinal tract, viruses and fungi also contribute to shaping the regional gut immune system and in the development of diseases. At the same time, dysregulation or abnormal colonization of the intestinal flora by pathogens including viruses and fungi, results in the development of many diseases, including those of the central nervous system, gastrointestinal tract, metabolism, etc. These diseases result from abnormal activation or inhibition of the intestinal immune system [10–12] and can negatively impact the intestinal microenvironment homeostasis by influencing the flora composition, possibly results in malnutrition [9]. In addition, the competitive relationships that occur among natural intestinal microflora are critical for maintaining microflora stability. For example, *Lactobacillus*, as well as other obligate anaerobes, produce acidic metabolites such as lactic acid, that create an acidic microenvironment not conducive to survival of aerobic conditioned pathogens [13]. Conversely, exploiting the notion that the intestinal flora and intestinal immune system affect each other, probiotics or microbial transplantation can be used to alter the intestinal microenvironment, ultimately contributing to the development of a healthy gut microbiota. This reshapes the immune homeostasis in the intestinal tract, and serves to treat or alleviate disease [14]. It is also possible to alter the intestinal microbiota by changing dietary habits, which can improve the microenvironment and immune function [15]. In short, the intestinal flora affects the development of disease by affecting the intestinal immunity. Further, by reshaping the intestinal microenvironment, the microflora improves the function of the regional immune system, resulting in disease relief and treatment.

In the current review, we provide an overview of the role played by intestinal microbiota within the regional intestinal immune system. Accordingly, we focus on the mutual impact between intestinal flora and intestinal area immunity, as well as the relationship between intestinal flora and disease. Finally, cutting-edge treatment strategies for intestinal flora are reviewed, while highlighting the recent advances and most pressing issues in the field.

## Effect of Intestinal Flora on Intestinal Area Immunity

### Intestinal Flora Promotes the Development of Intestinal Immune System

Development of the immune system exists in every stage of life. However, the influence exerted by the internal and external environment on shaping the early immune system is particularly important, especially before and immediately after birth.

**Figure 1:**
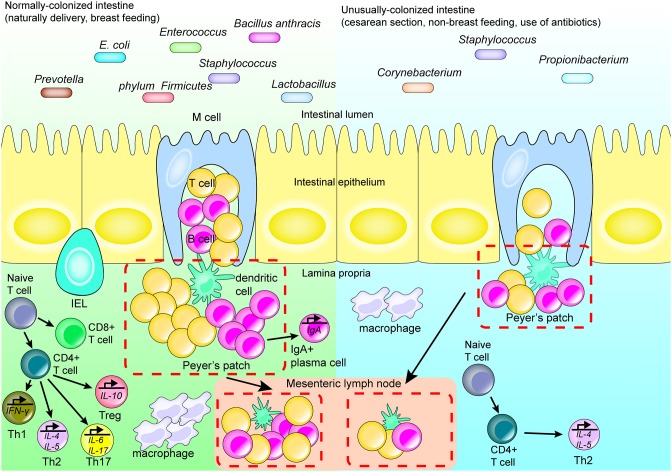
Intestinal flora affects the development of the regional immune system. Many factors affect the structure and development of the regional immune system by affecting the type and quantity of intestinal flora. Cesarean section, formula-feeding and the use of antibiotics after birth may lead to abnormally colonized intestines compared with fetuses delivered vaginally and breast fed. In a healthy normal intestinal tract with abundant microbial flora, the isolated lymphoid follicles and Peyer's patches in the intestine are unchanged. Upon stimulation, antigen-presenting cells migrate to the mesenteric lymph node and promote differentiation of T cells. IgA-producing plasma cells secrete IgA and perform a mucosal protective function; while macrophages migrate to the lamina propria of the intestinal tract and perform their normal function. When the species abundance and diversity of intestinal flora are relatively low, the intestinal immune system does not become appropriately stimulated and only isolated lymphoid follicles are formed.

In the early life stages of life, appropriate colonization by intestinal flora results in PAMP stimulation of PRRs expressed on the intestinal mucosal epithelial cells or immune cells. This stimulation subsequently induces maturation of the intestinal mucosa-associated lymphoid tissue [6]. Should a well-constructed gut flora fail to form in the early life stages, the immune function in the region is negatively affected. This is exemplified by incomplete development of the intestinal mucosa-associated lymphoid tissue, reduced number of immune cells and cytokines, and downregulation of surface PRR expression [16]. In fact, several studies have shown that maternal diseases and environmental contamination can alter the fetal intestinal microbiota composition; which can negatively impact development of the intestinal immune system [17, 18]. Furthermore, in cases of intestinal bacterium deficiency, the intestinal immune system is affected. For instance, in germ-free animal models, gut-associated lymphatic tissue deficiency is observed in the form of reduced numbers of IgA-producing plasma cells and intraepithelial lymphocytes (CD4^+^ T cells and CD8^+^ T cells; Figure 1) [19]. Also, within these animal models, the functionality of regulatory T cells (Tregs) is reduced [20, 21], the germinal center of Peyer's patches is small [22], and the development of isolated lymphoid follicles is inhibited [23]. Other studies have directly reported that intestinal bacteria promote the development of the immune system. For example, intestinal superantigen-like molecules of *Bacillus anthracis* promote development of B cells in Gut-Associated Lymphoid Tissue (GALT) [24].

The intestinal flora of a fetus contributes to immunity primarily at birth and after birth. Although the fetus is maintained in a relatively sterile environment inside the womb, it is exposed to certain bacteria, e.g., from the phylum Firmicutes and family Enterobacteriaceae, which affect the development of the fetal immune system, via the placenta, membrane, and cord blood [25–27]. For example, during pregnancy, the presence of certain bacterial proteins in the mother's gut, including metabolites of *Escherichia coli* HA107, elicit production of antibodies within the mother's serum. These antibodies then enter the fetal blood affecting early postpartum innate immunity and increasing production of fetal group 3 innate lymphoid cells (ILC3) as well as F4/80^+^ CD11c^+^ mononuclear cells [28]. However, upon birth, the newborn is rapidly exposed to microbes present in the environment, and colonization by the intestinal flora begins. This affects the development of the regional immune system, depending on the types of microbes present at birth [29]. *Lactobacillus, Prevotella*, and others are the primary types of bacteria colonizing a vaginally-delivered infant; alternatively, an infant delivered by cesarean section is primarily colonized by *Staphylococcus, Corynebacterium*, and *Propionibacterium* species (Figure 1) [30, 31]. In addition to these differences in the type of intestinal flora, the number of gut microbes in infants delivered by cesarean section is also lower than that of infants born via vaginal delivery [32], which has been found to be associated with pronounced decreases in immune tolerance, T-cell responses and production of specific cytokines. For example, the T-helper cell-related chemokines, CXCL10 and CXCL11, were reported as decreased in intestinal epithelial cells, with a corresponding negative impact on immune system development, in infants delivered via cesarean section compared with vaginally-delivered infants [33].

During the infant stage, various dietary and environmental factors also affect the intestinal flora composition, which in turn differently affects the initial development of the immune system [34]. Should stable and appropriate microbial colonization fail early in life, the development of the immune system throughout life becomes affected. For example, *Bifidobacterium* becomes dominant in early life and produces cell surface extracellular polysaccharides, positively affecting the development of mucosal immune cells [35]. At the same time, early colonization by microorganisms was shown in some animal experiments to promote the expression of CXCL16, a chemoattractant ligand on the intestinal epithelial cells, and to regulate the quantity and function of invariant natural killer T (iNKT) cells in the colon and lung [36, 37]. Th1 and Th2 cell responses, which are important components of adaptive immunity, are also impacted by intestinal microflora. In healthy individuals, these cells are in equilibrium; however in germ-free mice, the equilibrium is skewed toward Th2 differentiation. This results in a decline in the number of Th1 cells that promote cellular immune responses, and Th17 cells that mediate defenses against extracellular pathogens and autoimmune diseases (Figure 1) [38, 39].

Collectively, the host immune system development depends on the contact and interaction of microorganisms with the intestine in early life. In the absence of such interaction, development of the host's immune system is affected to a varying degree.

### Intestinal Flora Activates the Intestinal Immune System

An increasing number of studies report that the intestinal flora secretes a variety of metabolites and bacteriocins, with some bacteria also activating the immune system via the expression of specific antigens, adhesion to the epithelium, and interaction with PPRs. This leads to metabolic changes in the gut and other organs of the body as well as in the development of diseases, including cancer [9]. Indeed, while this effect protects the intestinal mucosa by maintaining balance in the regional immune microenvironment, it might also cause a series of diseases mediated by proinflammatory cytokines that consequently aggravate intestinal inflammation [9].

**Figure 2:**
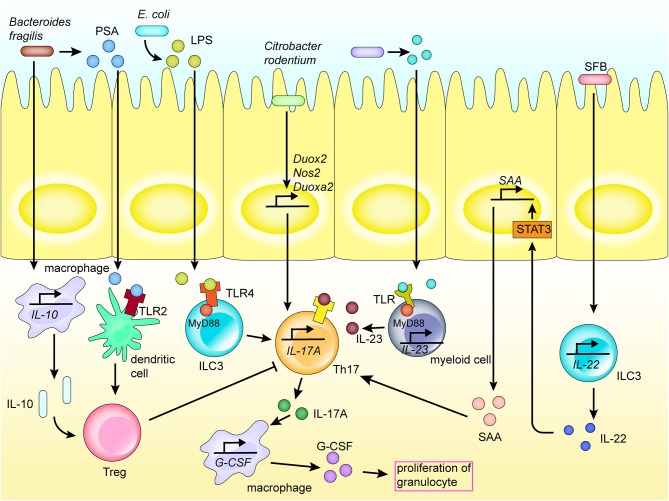
Mechanism of Th17 activation of the regional immune system in the intestinal tract. Th17 cells play an important role in the activation of the regional immune system induced by the intestinal flora. Bacteria and their products are recognized by receptors expressed on the surface of various immune cells by direct adhesion or translocation to the lamina propria, thereby inducing various cytokines and chemokines, and activating the intestinal mucosal immune system. Molecules recognized by TLR receptors promote the secretion of cytokines, hence promoting the differentiation of Th17 cells. Th17 cells secrete cytokines to promote the activation of inflammatory pathways in immune cells and recruit macrophages.

Adhesion to epithelial cells is an important route for direct microbial communication with the immune system. For instance, segmented filamentous bacteria adhere to epithelial cells, and induce the activity of CCR6^+^ ILC3s, which are distributed in isolated lymphoid follicles and crypts, to increase secretion of interleukin (IL) 22 in the gut. IL-22 induces the production of serum amyloid A (SAA) by the intestinal epithelial cells by activating the STAT3 pathway. Further, SAA directly acts on Th17 cells in the gut lamina propria to promote their differentiation (Figure 2) [40]. As an example, the bacterium *Citrobacter rodentium* contains a series of core virulence factors expressed by virulence genes in the Locus of Enterocyte Effacement. These factors are also expressed in human enteropathogenic *E. coli* and enterohemorrhagic *E. coli*, which may contribute to adoption of the colonization strategy of attaching and effacing (A/E) lesion formation [41, 42]. Further, the adhesion of *C. rodentium* to intestinal epithelial cells upregulates the expression of *Nos2, Duoxa2*, and *Duox2* genes involved in the production of reactive oxygen species, also promoting the differentiation of Th17 cells in the colonic lamina propria (Figure 2) [41].

Many myeloid cells in the intestine, such as macrophages and dendritic cells, are activated by the intestinal flora to initiate innate and adaptive immunity, and inflammatory reactions [43]. In addition, intestinal epithelial cells, as the first barrier of innate regional immunity, play an important role in activating the immune system and protecting the host from pathogens [44]. Flagellin of some gram-negative rod-shaped bacteria (such as *E. coli*) stimulates the Toll-like receptor (TLR) 5/MyD88 signaling pathway, promotes the production of IL-8 by epithelial cells, and recruits neutrophils to the lamina propria, triggering a series of regional immune responses [45].

The intestinal epithelium, and numerous natural immune cells, express a series of PRRs, such as TLRs and NOD-like receptors (NLRs). These receptors enable direct sensing of carbohydrates and lipid-based bacterial cell wall components, e.g., LPS, peptidoglycan, lipoteichoic acid, and other PAMPs, thereby activating the immune system [45]. For example, lipoproteins and LPS secreted by certain bacteria are recognized by TLR-1, 2, 4, 6, and 10 on the surface of myeloid cells, mucosal epithelial cells and other immunocytes [45, 46]. In fact, segmented filamentous bacteria, *Salmonella*, and the pathobiont *Proteus mirabilis*, among others, activate phagocytic cells via TLRs, and further induce the activation of Th17 and Th1 responses in adaptive immune cells, which then secrete proinflammatory cytokines, such as IL-1β, IL-6, and IL-23 (Figure 2) [47].

The recognition of TLRs activates downstream MyD88 and several inflammatory response pathways, such as mitogen-activated protein kinase/NF-κB, through a series of signaling cascades to promote the expression of cytokines, including IL-6 and TNF, leading to intestinal inflammation [48, 49]. For instance, *Fusobacterium nucleatum* produces virulence factors (such as FadA, Fap2, and LPS) that are recognized by TLRs (TLR2 and TLR4), leading to the activation of inflammatory pathways (e.g., NF-κB) and promoting production of proinflammatory cytokines (IL-1β, IL-6, IL-8, and TNF) and reactive oxygen species. This is followed by inflammatory response and DNA damage [50]. For instance, the TLR/MyD88 response pathway has been reported to induce expression of COX-2 in stromal cells, including macrophages, thereby regulating inflammatory responses and promoting angiogenesis, while also playing an important role in the development of inflammation and progression of colorectal cancer [51]. Further, Paneth cells are stimulated by specific bacterial virulence factors, such as adhesin produced by *E. coli*, which regulates the production of antibacterial molecules via the TLR4/MyD88 signaling pathway. Bacterial LPS is recognized by ILC3 TLR4, which then adheres to MyD88, inducing ILC-A to secrete IL-17A, which increases secretion of granulocyte-colony stimulating factor and promotes the increase of granulocyte levels [46, 52]. Further, *Salmonella typhimurium* activates the Wntβ-catenin signaling pathway by activating TLR4/MyD88 signaling, hence triggering inflammation [53]. Bacterial products are also sensed by TLRs on myeloid cells, which can cause activation of IL-23–producing myeloid cells. The resultant IL-23 promotes production of IL-17A, e.g., by Th17 (Figure 2) [43].

The TLRs/MyD88 response pathway can also promote cancer development by activating the regional immune system. The microbial population, specifically, promotes upregulation of IL-17C by intestinal epithelial cells via the TLR/Myd88-dependent signaling pathway. IL-17C induces expression of Bcl-2 and Bcl-xL in intestinal epithelial cells in an autocrine manner, to promote cell survival and tumorigenesis [54]. Further, *F. nucleatum* may impact colorectal cancer through TLR4 and TLR2. The stimulation of TLR2/4 results in a selective deletion of miR-18a^*^ and miR-4802 expression, leading to decreased expression of autophagy-related proteins ULK1 and ATG7, and upregulation of miR-34a expression, thereby increasing resistance of colorectal cancer cells to chemotherapy and subsequent progression of cancer [55, 56]. At the same time, peptidoglycan derived from gram-negative bacteria induces a large number of isolated lymphoid follicles by acting via NOD1 receptors in intestinal epithelial cells. Analysis of *Shigella flexneri* and *Helicobacter pylori* infection models revealed that in addition to TLR signaling, these bacteria promote NOD1 receptor activation in the NF-κB signaling pathway in epithelial cells, enhancing the killing ability of bone marrow neutrophils [56].

As for fungi, C-type lectin receptor Dectin-1 is an innate immune receptor that recognizes β-1,3-glucans in the fungi cell walls to activate macrophages and dendritic cells, which subsequently exert a killing effect on the fungi. This binding can also activate caspase recruitment domain-containing protein 9, and NF-κB intracellular signals to induce Th17 immune responses [57–59].

**Figure 3:**
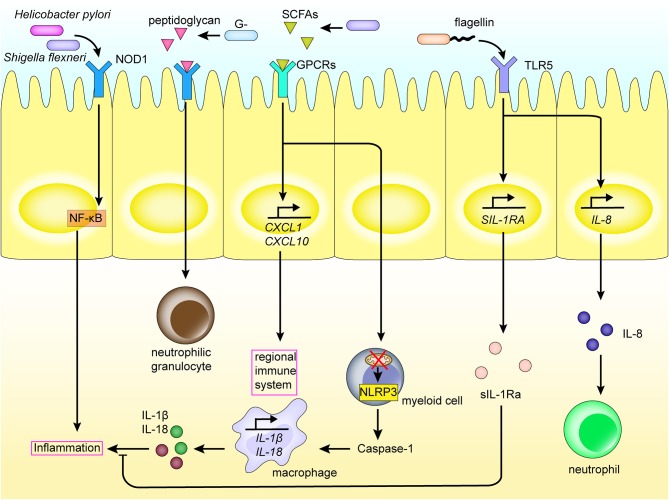
Intestinal flora and metabolites interact with the regional immune system in the intestinal tract. PRRs such as NOD1, TLRs expressed on the intestinal mucosal epithelium, recognize PAMPs and SCFAs of the intestinal flora, thereby activating inflammatory pathways and the production of inflammatory cytokines. This induces the differentiation of myeloid cells, recruitment of macrophages and neutrophils, and so on, elicits an inflammatory response, and activates the regional immune system. The interaction also promotes the expression of inflammatory factors. In addition, the intestinal flora also induces the production of cytokines in the intestinal mucosal epithelium through TLRs.

The metabolome is comprised of a set of metabolites synthesized by the host biological system, and includes products of both the host and microbial metabolism [60, 61]. The diverse metabolites produced by microbiota, such as polyamines, short-chain fatty acids (SCFAs) and AHR ligands may affect the immune response and disease progression through interacting with cells in the intestinal tract of the host [62]. For example, intestinal microbiota affect the host's regional immunity by digesting dietary fiber into SCFAs. Some SCFAs are then used as energy sources for cells, upon passive and active absorption by the intestinal epithelial cells [63]. However, others are used as signals activating the immune system and are recognized by G protein-coupled receptors (GPCRs) on the surface of intestinal epithelial cells, such as the free fatty acid receptor 2 (FFAR2) and FFAR3. This induces the production of cytokines and chemokines, including TNF-α, IL-6, CXC chemokine ligand-1 (CXCL1), and CXCL10, and activates the regional immune system (Figure 3) [64]. Moreover, butyrate and propionate from SCFAs have also been shown to inhibit development of dendritic cells via interaction with FFAR [65]. SCFA recognition by GPCRs on the epithelial cell surface also activates NACHT, Leucine-rich repeat, and Pyrin domain domain-containing protein 3 (NLRP3) production [66]. Toxic bacterial products also function to damage the mitochondria of myeloid cells, which in turn leads to production of the inflammasome, including NLRP3, in the cytoplasm of myeloid cells, and inducing production of caspase-1, IL-1β, and IL-18, causing an inflammatory reaction [67, 68]. Further, NLRP6 is capable of sensing microbiota metabolites such as taurine, which in turn activates cleavage of pro-IL-1β and pro-IL-18, as well as the production of IL-18. Increased levels of IL-18 subsequently affects the intestinal microbial composition and improves the development of dextran sodium sulfate (DSS) colitis. Alternatively, NLRP6 deficiency induces the production of CCL5 and immune cell recruitment, which could lead to spontaneous inflammation [69, 70].

In conclusion, activation of the intestinal immune system has both positive and negative effects on maintenance of human health. As one of the primary factors activating intestinal immunity, intestinal flora plays an important role in protecting the body from pathogens and promoting formation of intestinal inflammatory response.

### Relationship Between Intestinal Flora, Regional Immune Regulation, and Regional Immune Tolerance

Immune tolerance prevents the body's immune system from eliciting an immune response to the intestinal flora that dwells symbiotically within the body, while inhibiting progression of inflammatory response. Tregs play an important role in immune regulation and immune tolerance. For instance, *Bacteroides fragilis* contains capsular polysaccharide A, which can activate FoxP3^+^ CD4^+^ Tregs in the lamina propria by up-regulating the inhibitory cytokine, IL-10; which subsequently activates the TLR2 signaling pathway. Further, *B. fragilis* may alter the Treg/Th17 balance by counteracting the inflammatory response induced by LPS, thereby exerting an immunomodulatory effect [71].

TLRs also play an important role in the mechanism of regional immune regulation. For example, polysaccharide A of *B. fragilis* is recognized by TLR2 on the surface of dendritic cells or IL-10–producing T cells, promoting the production and differentiation of Tregs, and inhibiting production of Th17, thus regulating immunity (Figure 2) [72, 73]. In addition, flagellin is recognized by TLR5 of the intestinal epithelial cells and macrophages, which induces the expression of sIL-1Ra, thus limiting IL-1β-mediated inflammatory responses (Figure 3) [74]. Further, *F. nucleatum* selectively induces proliferation of bone marrow-derived immune cells, which promotes production of reactive oxygen species and inflammatory cytokines (such as IL-6, IL-10, and TNF) in colorectal cancer cells. This inflammatory response inhibits T-cell proliferation and induces T-cell apoptosis to regulate T-cell-mediated adaptive immunity [75]. Alternatively, *Enterococcus faecalis* inhibits macrophage phagocytosis by inhibiting macrophage production of NLRP3 inflammatory bodies in the presence of food source acetic acid, while simultaneously inhibiting the activation of mitogen-activated protein kinase signaling and, ultimately, inflammation [76].

## Intestinal Flora Affects Disease Development by Affecting Regional Immunity

### Disorders of Intestinal Flora Affect the Relationship Between the Immune System and Disease

The key direct effect induced by disorders of the intestinal microbiota is the destruction of immune system homeostasis in the intestinal tract, which leads to various gastrointestinal diseases [11, 12]. For instance, Liu et al. reported that *F. nucleatum* aggravates progression of ulcerative colitis [77]. Specifically, the bacterium induces M1 differentiation of macrophages in the intestinal mucosal lymphoid tissue; simultaneously, the Th1-associated cytokine IFN-γ is produced by the AKT2 signaling pathway, aggravating the disease [77]. Moreover, in colitis, the intestinal microbiota and specific microbial components activate downstream MyD88 and PI3K pathways through TLR2, to activate IL-10–producing B cells. IL-10 then acts on T cells to alleviate T-cell-mediated chronic colitis [78]. In a DSS–induced colitis mouse model, fungal antigens present in the gut lamina propria induced production of anti–*S. cerevisiae* antibodies and activated the immune system. However, if the receptor Dectin-1 was deficient, the fungal infection worsened and susceptibility to colitis increased [79]. Alternatively, *S. boulardii* has proven effective as a probiotic to contribute to the prevention of antibiotic-associated diarrhea through restoring the normal intestinal flora [80].

**Figure 4:**
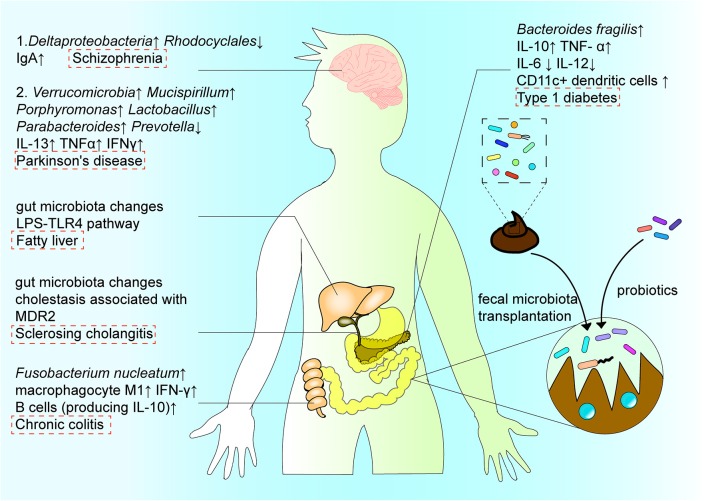
The interactions among intestinal flora, regional immune system, disease, and treatment. The structure and function of intestinal microorganisms have a duo-impact on disease by affecting the immune system in the intestinal region. In the digestive system, *Fusobacterium nucleatum* induces the production of cytokines IL-10 and IFN-γ, and the differentiation of macrophages to aggravate the progress of ulcerative colitis. In the nervous system, the increase of Deltaproteobacteria abundance and decrease in Rhodocyclales abundance affect the intestinal microenvironment, and subsequently affect the course of schizophrenia via the “gut–brain” axis. In metabolic diseases, *B. fragilis* aggravates the development of type 1 diabetes by changing the expression of cytokines. In addition, the intestinal microenvironment can be reconstructed by probiotics or microbiota transplantation, with typically occurring organisms reconstituted via fecal microbiota transplantation, so as to recalibrate immune homeostasis in the intestinal region, and contribute to the treatment and amelioration of disease.

In addition to intestinal diseases, other digestive diseases are affected by intestinal flora imbalance, which in turn alters the structure or function of intestinal immunity to drive disease development (Figure 4). As an example, Liao et al. demonstrated that MDR2-related cholestasis triggers intestinal microecological disorders in a mouse model of sclerosing cholangitis [81]. After observing changes in the intestinal flora, endotoxin produced by gut bacteria entered the portal vein to activate the NLRP3 inflammasome, leading to severe liver damage. This process did not depend on caspase-8 in hepatocytes, however, could be blocked by a pan-caspase inhibitor (IDN-7314) [81]. Abnormal changes in fungi can also be directly impacted by certain diseases. For example, in alcoholic liver disease, chronic alcohol consumption can lead to intestinal Candida overgrowth, which subsequently results in increased levels of β-glucans in the cell walls of circulating fungi, and reduced intestinal fungal diversity. This change can then induce an increase in IL-1β expression as well activation of Kupffer cells by C-type lectin domain family 7 member A, ultimately causing hepatocyte damage and liver inflammation [82].

Other diseases are also closely associated with gut microbes. For example, changes in the intestinal microbiota can exert a certain mitigating effect on viral damage of the nervous system. Indeed, intestinal microbes promote the CD4^+^ T cell immune response, both inside and outside of the intestine. Depletion of intestinal flora results in the inability of microglia to produce antigen-specific T-cell immunity, and the number of virus-specific CD4^+^ and CD8^+^ T cells is correspondingly reduced [83]. Further, loss of intestinal flora reduces the percentage of total microglia, the number of microglia cells expressing major histocompatibility complex (MHC) molecules, and co-stimulatory molecules, such as CD86 and CD40, required for antigen presentation. Indeed, the TLR ligands produced by intestinal flora are recognized by the intestinal immune system and trigger antigen presentation by microglia, contributing to TLR4 signal transduction in microglia, achieving the highest level of MHC-I and MHC-II upregulation to inhibit viral diseases [84, 85].

In addition, the gut microbiota may play an important role in the pathogenesis of schizophrenia via the “gut–brain” axis, by affecting the intestinal immune system. As an example, Xu et al. [10] reported significant changes in the types and levels of intestinal bacteria in individuals with schizophrenia, including increased abundance in *Deltaproteobacteria* species and decreased abundance in *Rhodocyclales* species, as well as changes in the intestinal immune environment, e.g., increased IgA levels (Figure 4). Moreover, the biomarkers of bacterial translocation, including lipopolysaccharide-binding protein and CD14, were reported to be significantly elevated in the serum of patients with schizophrenia. However, select probiotics have proven effective in reducing specific antibodies against pathogens reportedly increased in the serum of patients with psychiatric illnesses, thereby assisting in relieving the associated symptoms. This may imply that the gut microbiota, as well as the innate immune response, have important roles in the immune imbalance that occurs in schizophrenia [10].

Moreover, multiple sclerosis, a serious autoimmune disease, generally leads to progressive destruction of the central nervous system and an imbalance in intestinal immune homeostasis often caused by severe alterations in the intestinal flora. This has been demonstrated by experimental autoimmune encephalomyelitis, an animal model of multiple sclerosis, which induces changes in the intestinal flora and the immune system [86]. Further, multiple sclerosis has been shown to induce an initial increase, followed by a decrease in IL-17 and IFN-γ production, with simultaneous decrease, followed by gradual increase in *Axl* and suppressor of cytokine signaling 3 (SOCS3) expression. Analysis of the intestinal flora associated with multiple sclerosis revealed a significantly decreased abundance of *Prevotellaceae* NK3B31 group, which may serve as a biomarker for multiple sclerosis [87]. Further, the abundance of this bacteria was negatively correlated with serum IFN-γ levels, and positively correlated with *Axl* mRNA levels. The latter may have been induced by IL-17 and IFN-γ following disease-induced changes in the immune system [87]. At the same time, decreased abundance of *Faecalibacterium, Prevotella*, and *Anaerostipes* genera, as well as increased abundance of *Bifidobacterium* and *Streptococcus* were shown in multiple sclerosis [88]. Hence, intestinal flora plays an essential role in inhibiting pathogenic colonization and protecting intestinal barrier while also promoting the systemic immune response [89]. Intestinal flora can also stimulate the differentiation of T cells and monocytes to activate the innate and adaptive immune system [90]. Taken together, a decrease in the abundance of resident intestinal beneficial bacteria with an increase in the proportion of pathogens, ultimately alters the systemic inflammatory response, which then contributes to the pathological changes associated with multiple sclerosis [91].

*B. fragilis* is a member of the colonic symbiotic microbiota in humans and impacts the development of metabolic diseases. By administering *B. fragilis* to non-obese diabetic mice with type 1 diabetes, Sofi et al. [92] showed that *B. fragilis* aggravates type 1 diabetes via the immune pathway. Specifically, the bacterium inhibited expression of IFN-γ by T cells and promoted expression of Foxp3^+^ cells. It also promoted IL-10 and TNF-α production, and inhibited production of IL-6 and IL-12 (Figure 1). Changes in the microbial composition and function can also regulate metabolic homeostasis and fat browning induced by caloric restriction via the LPS-TLR4 pathway, and play a positive role in the treatment of fatty liver and other diseases [93].

Intestinal fungal and viral microbiota also contribute to the stability of the intestinal immune microecology. Fungal species, specifically, account for approximately 1% of the gut microbes [94]. However, with the development of DNA sequencing technology, additionally fungal species are constantly being identified as colonizers of the intestine, including *Candida albicans, Wallemia mellicola, Aspergillus*, and *Epicoccum* spp [95]. Moreover, fungal changes contribute to immune responses as well as the development of diseases such as IBD. Specifically, among IBD patients the proportion and abundance of fungal species has been reported to become altered, for example, in these patients, the Basidiomycota/Ascomycota ratio improved markedly while the proportion of *Saccharomyces cerevisiae* decreased and that of *C. albicans* increased [96].

Additionally, enterovirus surface antigen can be recognized directly by relevant intestinal mucosal immune cells through the cytosolic sensors, retinoic acid inducible gene I, melanoma differentiation–associated protein 5, endosomal TLRs, as well as PRRs, which function to induce further production of IFN [97]. Certain viruses, such as phages, are also closely associated with bacterial diversity, which indirectly plays a role in the intestinal immune system and contributes to development of disease. For example, in Crohn's Disease (CD) patients, Caudovirales viruses are reported as positively correlated with the proportion of Enterobacteriaceae and Pasteurellaceae, bacteria, all of which were increased in CD patients [98].

### Changes in the Regional Immune System and Intestinal Flora Are Induced by Homeostasis Imbalances

Imbalance in intestinal homeostasis may lead to characteristic changes in the immune system of the intestinal region and affect the microflora composition. Often, changes in the intestinal immune system and the intestinal flora are correlated. The intestinal microbiome and immune status of the intestinal tract change following parasitic infection. In such infections, the pathologic change associated with allergy, the formation of immune complex and the occurrence of inflammatory reactions are generally associated with the body's immune response to parasite eggs in the gastrointestinal or urinary system. These responses often lead to increased IL-4 and IL-5 production (a Th2 immune response) and, ultimately, granuloma formation. Concurrently, the abundance of IL-2 and IFN-γ (a Th1 immune response) decreases, thereby initiating the competition between Th1 and Th2 immune responses induced by intestinal microbiota. The Th2 response is triggered by schistosome eggs and is subsequently directed primarily against egg antigens; whereas the Th1 immune response is induced largely by larval antigens [99, 100]. Nevertheless, the composition of intestinal flora can change during parasite infection likely due to the parasite-induced changes occurring in the intestinal immune status. For example, urogenital schistosomiasis can alter the abundance of different groups of gut microbes by decreasing the abundance of *Firmicutes* and increasing *Proteobacteria*. In addition, changes in the intestinal flora are closely related to the inflammatory response, and include reduced abundance of *Clostridiales* and increased abundance of *Moraxellaceae, Veillonellaceae, Pasteurellaceae*, and *Desulfovibrionaceae*, eventually leading to an incompatibility between the bacterial flora and the immune system.

Disordered intestinal homeostasis is also associated with other diseases of the central nervous system. For instance, altered intestinal flora has been shown to correlate with disease severity and clinical phenotypes in Parkinson's disease. Such changes include the increase in *Verrucomicrobia, Mucispirillum, Porphyromonas, Lactobacillus*, and *Paraideides* abundance, and decrease in *Prevotella* (Figure 4) through the Microbiota-Gut-Brain axis. Dysfunction in the nervous system often leads to dysfunction in the GI tract, causing dysphagia, constipation, and increased intestinal permeability. The impaired gut barrier function can cause translocation of microorganisms and harmful products produced by the intestinal flora, such as LPS, leading to an abnormal immune response [101]. At the same time, the abnormal immune response can lead to inflammation during progression of Parkinson's disease, which is associated with increased levels of inflammation factors, such as IL-13, TNF-α, and IFN-γ [102]. The correlation between *Bacteroidetes* and plasma TNF-α levels, as well as between *Verrucomicrobia* and plasma IFN-γ levels, reflect the association between Parkinson's disease and the systemic inflammatory response, as well as the interaction between the disease and altered intestinal flora [102].

## Intestinal Flora can be Used to Reconstruct Regional Immunity and Treat Diseases

By exploiting the mutual relationship between the intestinal flora and immunity, the regional immunity can be reconstructed by changing the composition and structure of intestinal flora to improve treatment of diseases (Figure 4). The mechanism by which intestinal flora affects the regional immune system includes secretion of metabolic products [6], expression of PAMPs [9], and prevention of pathogen adhesion [103]. For example, probiotics including *bifidobacteria* and *lactobacilli*, affect autoimmunity [104]. This has been supported by a study that fed non-obese diabetic mouse a xylooligosaccharide diet and observed a subsequent reduction in intestinal marker permeability, primarily from M1 to M2 macrophages, an increase in the activation of regulatory T cells, and a reduction in the levels of activated cytotoxic T cells and NKT cells. A more pronounced regional and systemic anti-inflammatory environment was also observed compared with the control group [105–110]. Hence, this treatment may serve to improve gut barrier and, thus, has the potential to activate autoreactive immune responses [104].

Further, probiotics can affect immune microenvironment homeostasis in the intestinal region and, thus, positively impact disease treatment. Chen et al. [111] reported certain changes in the intestinal microflora and inflammatory cytokine levels in healthy pregnant women who received probiotics for an average of 7 weeks. Compared with a control group, the proportion of highly abundant taxa and core microbiota changed in the probiotic-using group; e.g., the abundance of *Streptococcus, Clostridium sensu stricto*, and *Ruminococcaceae* has significantly increased, while that of *Turicibacter* and *Phascolarctobacterium* significantly decreased. Further, the levels of IL-5, IL-6, TNF-α, and granulocyte-macrophage colony-stimulating factor were significantly increased following probiotic use. These results suggest that probiotics contribute to the transformation of an anti-inflammatory to proinflammatory status, and may affect the development of certain diseases. The use of probiotics has been shown to be important during pregnancy and may effectively modulate the immune system to one predominated by a proinflammatory state in the third trimester [111].

Besides, probiotics have a beneficial therapeutic role in inflammatory bowel disease. As shown by Rodriguez-Nogaleset et al. [112], the probiotic *Saccharomyces boulardii* increases intestinal bacteria diversity and alleviates the ecological imbalance of intestinal flora, so as to ameliorate and control the complex pathogenesis of inflammatory bowel disease. In terms of the underpinning immune mechanism, *S. boulardii* reduces the production of proinflammatory factors IL-1β and transforming growth factor β, thus inhibiting the inflammatory response. *S. boulardii* also regulates the levels of certain miRNAs that show abnormal expression in disease. For example, this yeast species inhibits expression of miR-155 and miR-223, which are increased in inflammatory bowel disease. Perhaps these changes in miRNA levels also play a role in the alleviation of diseases [112]. As another example, *Lactobacillus plantarum* ZLP001 impacts the composition of the intestinal microflora, and increases the abundance of butyrate-producing bacteria such as *Anaerotruncus* and *Faecalibacterium* in the 30-day trial [113]. Meanwhile, in terms of immune regulation, the strain downregulates production of proinflammatory cytokines, including IL-6, IL-8, and TNF-α, and increases levels of host defense peptides pBD2 and PG1-5. Ultimately, *L. plantarum ZLP001* strengthens the intestinal barrier by improving the epithelial defense function and regulating the intestinal microbiota, which reduces the risk of infection [113].

In addition to exerting a positive effect on diseases in the intestinal region, probiotics also play a certain role in the treatment of multiple sclerosis, by regulating the composition of intestinal flora. Within the intestinal microbiota, the use of probiotics reduces pathogenic bacterial growth during multiple sclerosis, including *Akkermansia* and *Blautia*, and increases the abundance of several groups that are depleted during the disease, such as *Lactobacillus* and *Bifidobacterium*. Meanwhile within the immune system, probiotics reduce the frequency of peripheral inflammatory monocytes, which can inhibit the expression of CD80 and HLA-DR on myeloid-derived dendritic cells (CD45^+^ Lin^−^ CD11C^+^), as well as the production of Th1 and Th17 cells. However, the expression of anti-inflammatory genes *Il10ra, Lilrb2*, and *Cybb* increases after probiotic use, while the expression of proinflammatory genes *Malt1* and *Lgals3* decreases [114].

The use of certain probiotics, such as *Lactobacillus reuteri*, can also alleviate immune checkpoint inhibitor-associated colitis caused by immunotherapy. The ability of *L. reuteri* (a 3-day treatment) to protect the colon is primarily associated with its ability to lower serum levels of anti-inflammatory factors, such as TNF-α, IFN-γ, and IL-6, as well as the ability to suppress serum levels of ILC3, IL-23, and IL-17, effectively relieving colitis induced by immunotherapy [115].

According to clinical studies, the use of antibiotics in infancy affects development of intestinal microflora and the intestinal immune system, and is associated with increased risk of asthma in children. Specifically, the use of prenatal antibiotics is associated, in a dose-dependent manner, with increased risk of asthma in children [116]. Further, babies delivered by cesarean section are more likely to suffer from chronic inflammatory diseases than babies born by vaginal delivery, which may be associated with altered colonization by intestinal microorganisms that activate the early immune system [117]. Regarding the specific mechanism, upon cesarean section, T cell numbers decrease while iNKT cell numbers increase compared with the level in natural labor. Simultaneously, the levels of regulatory markers, including Foxp3, IL-10, Ctla4, the macrophage markers Cd11c, Egr2, and Nos2 are downregulated, while those of iNKT cell markers IL-4 and IL-15 are increased. Hence, it may be possible to prevent diseases by altering the microbial colonization of the infant gut [117].

Healthy intestinal flora maintains immune homeostasis in the intestine by a variety of mechanisms, to protect the intestinal tract from diseases. Therefore, fecal microflora transplantation (FMT) is considered to be a promising approach for the treatment of existing diseases. For instance, FMT reduces proinflammatory cytokine levels, including those of TNF, IL-1β, and IFN-γ, and activates different immune mediated pathways, with a concomitant reduction of colonic inflammation and initiation of intestinal homeostasis recovery [14]. Further, FMT significantly increases MHC-II–dependent secretion of IL-10 by CD4^+^ T cells and is useful for the treatment of experimental colitis, while also reducing the ability of dendritic cells, monocytes, and macrophages to present MHC-II–dependent bacterial antigens to colonic T cells [14]. For example, *Lactobacillus fermentum* reduces the levels of proinflammatory cytokines, such as IL-2, IFN-γ, IL-4, IL-13, and IL-17A, while increasing levels of anti-inflammatory cytokines, such as IL-10, to relieve symptoms of inflammatory bowel disease [118]. Further, FMT alleviates antibiotic-related depletion of IL-33 during colonic inflammation caused by *Clostridium difficile* [117]. IL-33 drives the activation of colonic group 2 natural lymphocytes (ILC2) during infection to prevent and relieve the severity of *C. difficile* infection.

Intriguingly, in inflammatory bowel disease, the CD risk allele ATG16L1 T300A impacts intestinal microbes and the immune system. Transplantation of human fecal biota into mice with mutated ATG16L1 T300A genes resulted in increased levels of *Bacteroides ovatus*, Th1 cells, and IL-23 [119]. In turn, IL-23 induced Th17 differentiation. These observations indicate that ATG16L1 T300A leads to ecological imbalance and immune infiltration before the onset of disease symptoms. FMT was also shown to improve the condition of HIV-infected patients by manipulating the human intestinal bacteria, however, the mechanism remains to be studied [120].

## Conclusions

The presence of specific bacteria, related metabolites, and specific immune cells can be used for early diagnosis of certain diseases. At the same time, FMT can be used to alter the composition of intestinal flora and treat diseases, including gastrointestinal diseases and diseases of the central nervous system. However, the specific mechanisms by which the intestinal flora affects the intestinal immune system, or the imbalance of intestinal flora affects disease states, are not yet fully characterized. Furthermore, before treating the analysis of intestinal flora as a proxy for disease diagnosis and treatment is possible, specific problems must be resolved. First, detailed exploration of the relationship between the host and intestinal flora is needed to identify specific intestinal flora that is highly associated with specific diseases. Second, improved means for regulating the intestinal flora are needed. The currently used FMT and probiotics are effective to some extent, however, their effects cannot be precisely controlled, which can limit the use of intestinal flora to maintain health and control disease. Further, it remains to be determined which diseases are best suited to treatment by alteration of gut microbe composition for optimal therapeutic results. In terms of changes in the regional immune system, the causal relationship between intestinal flora and the altered regional immune system with changing health status in human should be clarified. Also, the understanding of the potential components and mechanisms that promote the interaction between the immune system and microorganisms should be improved. Nonetheless, we predict that in the future, the microbiome will become a powerful therapeutic tool enabling customized treatment, involving personalized microorganisms and microenvironments, and that early diagnosis of a variety of diseases based on monitoring of the microbiome and immune system will be possible. This will have a profound impact on the improvement of human health and development of precision medicine.

## Author Contributions

BZ and YY wrote the manuscript. BZ, SZ, and CG made the figures. XL, GL, WX, and ZZ revised the manuscript. ZZ designed the manuscript. All authors approved the final manuscript.

### Conflict of Interest

The authors declare that the research was conducted in the absence of any commercial or financial relationships that could be construed as a potential conflict of interest.
